# Enantiospecific Effects of Ketoconazole on Aryl Hydrocarbon Receptor

**DOI:** 10.1371/journal.pone.0101832

**Published:** 2014-07-07

**Authors:** Aneta Novotna, Martina Korhonova, Iveta Bartonkova, Anatoly A. Soshilov, Michael S. Denison, Katerina Bogdanova, Milan Kolar, Petr Bednar, Zdenek Dvorak

**Affiliations:** 1 Regional Centre of Advanced Technologies and Materials, Faculty of Science, Palacky University, Olomouc, Czech Republic; 2 Department of Environmental Toxicology, University of California Davis, Davis, California, United States of America; 3 Department of Microbiology, Faculty of Medicine and Dentistry, Palacky University, Olomouc, Czech Republic; Kobe University, Japan

## Abstract

Azole antifungal ketoconazole (KET) was demonstrated to activate aryl hydrocarbon receptor (AhR). Since clinically used KET is a racemic mixture of two *cis*-enantiomers (2R,4S)-(+)-KET and (2S,4R)-(−)-KET, we examined the effects of KET enantiomers on AhR signaling pathway. (+)-KET dose-dependently activated AhR in human gene reporter cell line AZ-AHR, and displayed 5–20× higher agonist activity (efficacy), as compared to (−)-KET; both enantiomers were AhR antagonists with equal potency (IC_50_). Consistently, (+)-KET strongly induced CYP1A1 mRNA and protein in human HepG2 cells, while (−)-KET exerted less than 10% of (+)-KET activity. In primary human hepatocytes, both enantiomers preferentially induced CYP1A2 over CYP1A1 mRNA and protein, and the potency of (+)-KET was slightly higher as compared to (−)-KET. Ligand binding assay with guinea pig liver cytosols revealed that both (+)-KET and (−)-KET are weak ligands of AhR that displaced [^3^H]-TCDD with comparable potency. Similarly, both enantiomers weakly transformed AhR to DNA-binding form with similar potency, as showed by EMSA, in guinea pig liver cytosolic extracts and nuclear extracts from mouse Hepa-1 cells. We also examined effects of KET on glucocorticoid receptor (GR), a regulator of AhR activity. Both KET enantiomers antagonized GR with similar potency, as revealed by gene reporter assay in AZ-GR cell line and down-regulation of tyrosine aminotransferase mRNA in human hepatocytes. Finally, we demonstrate enantiospecific antifungal activities of KET enantiomers in six *Candida spp*. strains. In conclusion, the significance of current study is providing the first evidence of enatiospecific effects of *cis*-enantiomers of ketoconazole on AhR-CYP1A pathway.

## Introduction

Ketoconazole (KET), azole antifungal agent acts as an inhibitor of enzymes involved in the synthesis of steroids. In particular, KET inhibits lanosterol 14 α-demethylase (CYP 51), which is necessary to convert lanosterol to ergosterol which leads to the accumulation of 14-α-methylsterols and inhibition of fungal growth. Furthermore, KET inhibits various enzymes involved in adrenal cortisol synthesis, and has been effectively used in the treatment of hypercortisolemia such as Cushing's syndrome [1]. KET also inhibits the synthesis of testosterone in both testicular and adrenal cells and therefore is used for treatment of androgen-dependent diseases such as advanced prostate cancer [2]. Despite the wide and frequent use of KET, there is much evidence of hepatotoxicity and hepatic tumors associated with it [3]–[5]. KET is extensively metabolized by the hepatic biotransformation enzymes [6]. KET has two chiral centers, therefore it exists in four enantiomers. The therapeutically active form of KET is a racemic mixture consisting of two *cis*-enantiomers; (2R,4S)-(+)-KET and (2S,4R)-(−)-KET. Two other *trans*-enantiomers were synthesized by Rotstein et al. and evaluated for their selectivity in inhibiting a number of cytochrome P-450 enzymes [7]. It was shown that *cis*-enantiomers are more potent inhibitors of 14-α-demethylase than trans-enantiomers. The largest difference in effects of individual *cis*-enantiomers was found for inhibition of progesterone 17α,20-lyase, when (+)-KET was a 40 times more potent inhibitor than (−)-KET [7]. KET inhibits several P450 enzymes, including CYP2C9, CYP2C19 and CYP3A4, resulting in potential drug-drug interactions with other medications [8]–[10]. Stresser et al. demonstrated that inhibition parameters of separate enantiomers for CYP3A4 are substrate-dependent and the data should be interpreted with care [11]. Nevertheless, enantioselective inhibition of CYP3A4 and CYP3A5 was reported [8]. KET also antagonized human glucocorticoid receptor (GR) [12] and displayed partial agonism towards pregnane X receptor (PXR), hence its effects on drug-metabolizing pathways are very complex [13].

It was described that KET and other antifungal drugs are inducers of CYP1A genes in human and murine cancer cell lines, tentatively through an AhR-dependent mechanism [14]. Since KET is a mixture of two *cis*-enantiomers, we carried out the study on stereospecific effects of KET *cis*-enantiomers, i.e. (2R,4S)-(+)-KET and (2S,4R)-(−)-KET on AhR-CYP1A signaling pathway. We measured transcriptional activity of AhR (and GR) using gene reporter assay. The expression of CYP1A1/2 mRNA and protein was evaluated in human hepatoma cell line HepG2 and in primary human hepatocytes. In addition, ligand binding assay and electromobility shift assay (EMSA) were performed to assess the ability of KET enantiomers to bind to and transform AhR. Overall, the novelty of current study is bringing the evidence on enantiospecific effects of KET on the AhR signaling pathway.

## Materials and Methods

### Compounds and reagents

Dimethylsulfoxide (DMSO), hygromycin B, dexamethasone (DEX) and ketoconazole were purchased from Sigma-Aldrich (Prague, Czech Republic). *Cis*-enantiomers of ketoconazole (2R, 4S)-(+)-KET and (2S, 4R)-(−)-KET were isolated from commercial ketoconazole by preparative HPLC at Department of Analytical Chemistry, Faculty of Science, Palacky University Olomouc. 2,3,7,8-tetrachlorodibenzo-*p*-dioxin (TCDD) was from Ultra Scientific (RI, USA). Luciferase lysis buffer was from Promega (Hercules, CA). All other chemicals were of the highest quality commercially available.

### Cell cultures

Human Caucasian hepatocellular carcinoma cells HepG2 (ECACC No. 85011430) and Human Negroid cervix epitheloid carcinoma cells HeLa (ECACC No. 93021013) were purchased from *European Collection of Cell Cultures (ECACC)* and cultured in Dulbecco's modified Eagle's medium (DMEM) supplemented with 10% of fetal bovine serum, 100 U/ml streptomycin, 100 µg/ml penicillin, 4 mM L-glutamine, 1% non-essential amino acids, and 1 mM sodium pyruvate. Cells were maintained at 37°C and 5% CO_2_ in a humidified incubator.

Primary human hepatocytes used in this study were obtained from two sources: (i) from multiorgan donors LH52 (female; 60 years) and LH54 (male; 71 years), when: a) Tissue acquisition protocol was in accordance with the requirements issued by “*Ethical Committee of the Faculty Hospital Olomouc, Czech Republic*” and with Transplantation law #285/2002 Sb., b) “*Ethical Committee of the Faculty Hospital Olomouc, Czech Republic*” approved the study and the use of primary human hepatocytes for research presented in the current paper., c) The consent from the next of kin of the donor was waived by “*Ethical Committee of the Faculty Hospital Olomouc, Czech Republic*”; (ii) long-term human hepatocytes in monolayer Batch HEP220770 (female; 35 years), Batch HEP220774 (female; 66 years) (purchased from Biopredic International, Rennes, France). Cells were cultured in serum-free medium. Cultures were maintained at 37°C and 5% CO_2_ in a humidified incubator.

### mRNA determination and quantitative reverse transcriptase polymerase chain reaction

Total RNA was isolated using *TRI Reagent* (Molecular Research Center, Cincinnati, OH, USA). cDNA was synthesized from 1000 ng of total RNA using M-MLV Reverse Transcriptase (Finnzymes, Espoo, Finland) at 42°C for 60 min in the presence of random hexamers (Takara, Shiga, Japan). qRT-PCR was carried out using LightCycler FastStart DNA MasterPLUS SYBR Green I (Roche Diagnostic Corporation, Prague, Czech Republic) on a Light Cycler 480 II apparatus (Roche Diagnostic Corporation). CYP1A1, CYP1A2, tyrosine aminotransferase (TAT) and GAPDH mRNAs were determined as described previously [15], [16]. Measurements were performed in triplicates. Gene expression was normalized to GAPDH as a housekeeping gene.

### Protein detection and Western blotting

Total protein extracts were prepared from cells on 6-well plates. Cells were washed twice with ice-cold PBS and scraped into 1 ml of PBS. The suspension was centrifuged (4500 RPM/5 min/4°C) and the pellet was resuspended in 150 µl of ice-cold lysis buffer (150 mM NaCl; 10 mM Tris pH 7.2; 0.1% (w/v) SDS; anti-protease cocktail, 1% (v/v) Triton X-100; anti-phosphatase cocktail, 1% (v/v) sodium deoxycholate; 5 mM EDTA). The mixture was vortexed and incubated for 10 min on ice and then centrifuged (15000 RPM/13 min/4°C). Supernatant was collected and the protein content was determined by the Bradford reagent. SDS–PAGE gels (10%) were run on a BioRad apparatus according to the general procedure followed by the protein transfer onto PVDF membrane. The membrane was saturated with 5% non-fat dried milk for 1 h at room temperature. Blots were probed with primary antibodies against CYP1A1 (goat polyclonal, sc-9828, G-18, dilution 1∶500), CYP1A2 (mouse monoclonal, sc-374228, G-4, dilution 1∶2000), actin (goat polyclonal; sc-1616, 1–19, dilution 1∶2000), all purchased from Santa Cruz Biotechnology (Santa Cruz, CA, USA). Chemiluminescent detection was performed using horseradish peroxidase-conjugated secondary antibodies (Santa Cruz Biotechnology) and Western blotting Luminol kit (Santa Cruz Biotechnology).

### EROD assay

HepG2 cells were plated on 96-well dishes at a density of 3×10^4^ cells per well in culture medium supplemented with 10% FBS and stabilized for 24 h. Cells were treated for 24 h with tested compounds. The catalytic activity of 7-ethoxyresorufin-*O*-deethylase (EROD) in cell cultures was measured as described elsewhere (Donato et al. 1993). Briefly, monolayers were washed with PBS and the medium containing 8 µM 7-ethoxyresorufin and 10 µM dicumarol was applied to the cells. After 30 min of incubation at 37°C, an aliquot of 75 µl of the medium was mixed with 125 µl of methanol and fluorescence was measured in 96-well plate with 530 nm excitation and 590 nm emission filters. The formation of resorufine was linear up to 60 min. The data were expressed as the ratio of treated over control values (DMSO-treated cells).

### Gene reporter assay and cytotoxicity

A stably transfected gene reporter cell line AZ-AHR, derived from human hepatoma HepG2 cells transfected with a construct containing several AhR binding sites upstream of a luciferase reporter gene, was used for assessment of AhR transcriptional activity [17]. A stably transfected gene reporter cell line AZ-GR, derived from human cervix carcinoma HeLa cells transfected with a construct containing several GR response elements upstream of a luciferase reporter gene, was used for assessment of GR transcriptional activity [18]. Cells were incubated for 24 h with tested compounds and/or vehicle (DMSO; 0.1% v/v), in the presence or absence of TCDD (5 nM; AZ-AHR cells) or DEX (100 nM; AZ-GR cells). After the treatments, cells were lysed and luciferase activity was measured. In parallel, cell viability was determined by conventional MTT test.

### Electrophoretic mobility shift assay

Electrophoretic mobility shift assay was performed in guinea pig cytosol and nuclear extract from mouse hepatoma cells. Hepa-1c1c7 cells were incubated for 2 h with tested compounds. Nuclear extracts procedure and EMSA analysis was performed as previously described (Soshilov & Denison; 2014). Guinea pig hepatic cytosol (8 mg/mL in HEDG) was incubated for 2 h in a room temperature water bath with tested compounds. An aliquot of the reaction was mixed with poly[dI•dC] and [^32^P]-DRE (100.000 cpm), and AHR:DRE:[^32^P]DRE complexes were resolved by gel retardation analysis, visualized by autoradiography and quantified by phosphorimager analysis (FujiFilm) of the dried gels [19].

### Ligand binding assay

[^3^H]-TCDD was kindly provided by Dr. Steven Safe (Texas A&M University) and 2,3,7,8-tetrachlorodibenzofuran (TCDF) was from Accustandard (New Haven, CT, USA). The competitive displacement of [^3^H]-TCDD from guinea pig hepatic cytosol was as previously described (Korashy et al., 2011). Briefly, hepatic guinea pig cytosol diluted to 8 mg/mL protein in MEDG (25 mM MOPS-NaOH, pH 7.5, 1 mM EDTA, 1 mM DTT, 10%, v/v glycerol) was incubated with (+)-KET, (−)-KET and rac-KET at concentrations 10 µM, 30 µM and 50 µM or 200 nM TCDF for 1 h at room temperature in the presence of 2 nM [^3^H]-TCDD. The amount of [^3^H]-TCDD specific binding was determined by hydroxyapatite protocol, and specific binding was determined as the difference between the ‘no competitor’ and TCDF reaction (Denison et al., 2002). [^3^H]-TCDD specific activity was 9.5 Ci/mmol. Non-specific binding was approximately 30% of total binding, i.e. specific binding was approximately 70% of total binding.

### MIC assessment

Antifungal activity was assessed using the standard microdilution method determining the minimum inhibitory concentrations (MICs) of the tested samples leading to inhibition of yeast growth. Disposable microtiter plates were used for the tests. The samples were diluted in brain heart infusion broth (HiMedia). For antifungal assays, the following *Candida* spp. strains were used: *C. albicans*, *C. krusei*, *C. tropicalis* and *C. parapsilosis*. To prepare a yeast suspension, 2–3 yeast colonies (following 12-hour incubation on Sabouraud agar) were inoculated into 2 mL of Mueller Hinton broth and incubated (1 h/37°C/5%CO2). The tested samples were diluted with the medium to a concentration of 100 µM. Subsequently, 100 µL were transferred into the first microplate well and serially diluted by a factor of 2. The prepared microplates were inoculated with a standard amount of the tested *Candida* spp. – the inoculum density in a well was equal to 10^5^ CFU/mL. The MIC was read after 48 hours of incubation at 37°C as the minimum inhibitory concentration of the tested substance that inhibited the growth of the *Candida* spp. strains. The minimum fungicidal concentration (MFC) is the minimum concentration of the sample required to kill the yeast after a defined period of incubation. The MFCs were determined by the inoculation method. With an applicator, 10 µL were transferred from the microplate wells with defined concentrations of the tested sample and inoculated onto Sabouraud agar (Trios). The MFC was determined as the lowest concentration that inhibited the visible growth of the used yeast. For growth curve assessment, the samples were serially diluted in 96-well microtiter plates. The plates were then inoculated with suspensions of the *Candida* spp. strains, covered with foil to prevent evaporation and moved to a spectrophotometer with a built-in incubator. Absorbance (630 nm) was measured every hour during 24 hours and the obtained values were used to plot growth curves

### Statistics

Experiments in cell cultures were performed at least four times, with each experiment performed in triplicates. For measurement of luminescence (luciferase activity) and absorbance (MTT), triplicates from each sample were run. One-way analysis of variance followed by Dunnett's multiple comparison post hoc test or Student's *t* test was used for statistical analysis of data.

## Results

### Effects of ketoconazole enantiomers on transcriptional activity of aryl hydrocarbon receptor AhR in human gene reporter cell line AZ-AHR

**Figure 1:**
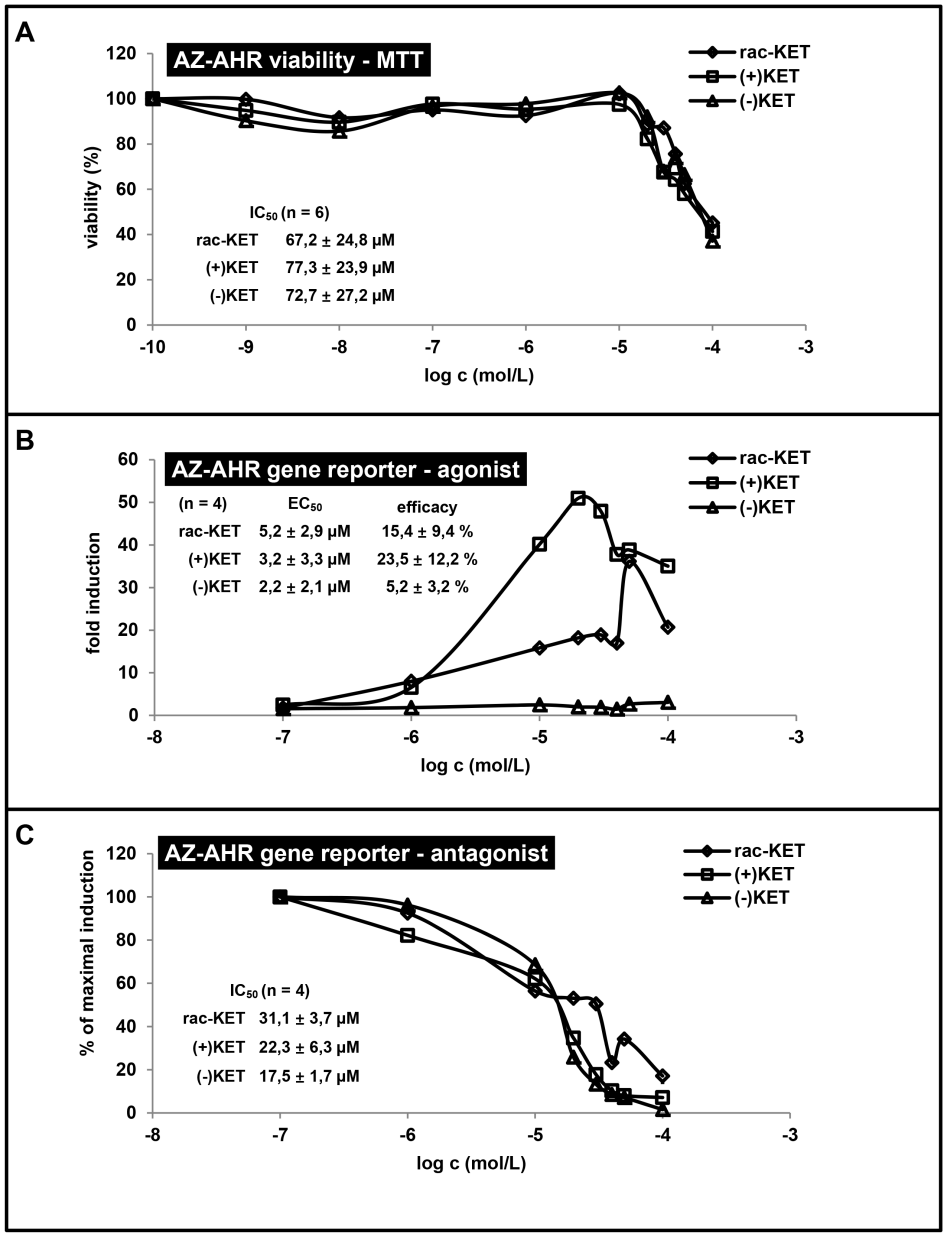
Effects of ketoconazole enantiomers on transcriptional activity of aryl hydrocarbon receptor AhR in human gene reporter cell line AZ-AHR. The cells were seeded in 96-well plates and stabilized for 16 h. **Panel A:** Cells were incubated for 24 h with (+)-KET, (−)-KET and rac-KET at concentrations ranging from 10^−10^ M to 10^−4^ M. The vehicle was DMSO (0.1% v/v). After the treatment, MTT test was performed and absorbance was measured at 540 nm. Treatments were performed in triplicates. The data are the mean from experiments from six different passages of cells and are expressed as a percentage of viability of control cells. The values of IC_50_ were calculated and are indicated in figure. **Panel B and Panel C:** AZ-AHR cells were incubated for 24 h with (+)-KET, (−)-KET and rac-KET at concentrations ranging from 10^−7^ M to 10^−4^ M in the absence (Panel B - *agonist mode*) or in the presence (Panel C - *antagonist mode*) of TCDD (5 nM). The vehicle was DMSO (0.1% v/v). After the treatments, cells were lysed and luciferase activity was measured. Treatments were performed in triplicates in four independent cell passages. Representative gene reporter assays are showed (from passage #4). Data are expressed as a fold induction of luciferase activity over control cells (Panel A - *agonist mode*) or as a percentage of maximal induction attained by TCDD (Panel B - *antagonist mode*). The values of EC_50_ and IC_50_ were calculated and the average values are indicated in figures.

Prior to the agonist or antagonist experiments, the cytotoxicity of tested compounds was assessed using the conventional MTT assay. For this purpose, AZ-AHR cells were incubated for 24 h with (+)-KET, (−)-KET and commercial rac-KET at concentration ranging from 100 pM to 100 µM. The vehicle was DMSO (0.1% v/v). After the treatment, a MTT test was performed. The values of IC_50_ were 77.3±23.9 µM, 72.7±27.29 µM and 67.2±24.8 µM for (+)-KET, (−)-KET and rac-KET, respectively (Figure 1A; showed representative plot from passage #4). There were no significant differences between the cytotoxicity of (+)-KET, (−)-KET and rac-KET.

Gene reporter assays were performed in two different experimental layouts. In *agonist mode*, cells were treated with increasing concentrations of tested compounds, and the half-maximal effective concentrations (EC_50_) were calculated. In *antagonist mode*, cells were incubated with increasing concentrations of tested compounds in combination with model agonist (TCDD; 5 nM), and half-maximal inhibitory concentrations (IC_50_) were calculated.

An induction of AhR-dependent luciferase activity by 5 nM TCDD in four consequtive passages of AZ-AHR cells varied from 366-fold to 613-fold (average induction 435-fold), as compared to vehicle-treated cells. Enantiomer (+)-KET strongly, dose-dependently activated AhR in concentrations up to 20 µM, with EC_50_ = 3.2 µM, and efficacy 10%–34% of TCDD at concentration 20 µM as compared to 5 nM TCDD (Figure 1B). At higher concentrations, luciferase activity declined likely due to the cytotoxicity (Figure 1A) and AhR-unrelated effects. On the other hand, only weak induction of luciferase activity was observed for (−)-KET, with EC_50_ = 2.2 µM, and efficacy 1%–8% at concentration 20 µM as compared to 5 nM TCDD (Figure 1B). Racemic commercial KET showed average effects of the combination of both (+)-KET and (−)-KET. TCDD-inducible transcriptional activity of AhR was dose-dependently inhibited by all forms of KET, with IC_50_ values of 22.3±6.3 µM, 17.5±1.7 µM and 31.1±3.7 µM for (+)-KET, (−)-KET and rac-KET, respectively. Unlike agonist activity, the inhibitory effects of KET on AhR were not enantiospecific (Figure 1C). Overall, we observed significant enantiospecific differences in agonistic, but not antagonistic effects of (+)-KET and (−)-KET on AhR.

### Effect of ketoconazole enantiomers on CYP1A1 mRNA, protein and EROD activity in human cancer cell line HepG2

**Figure 2:**
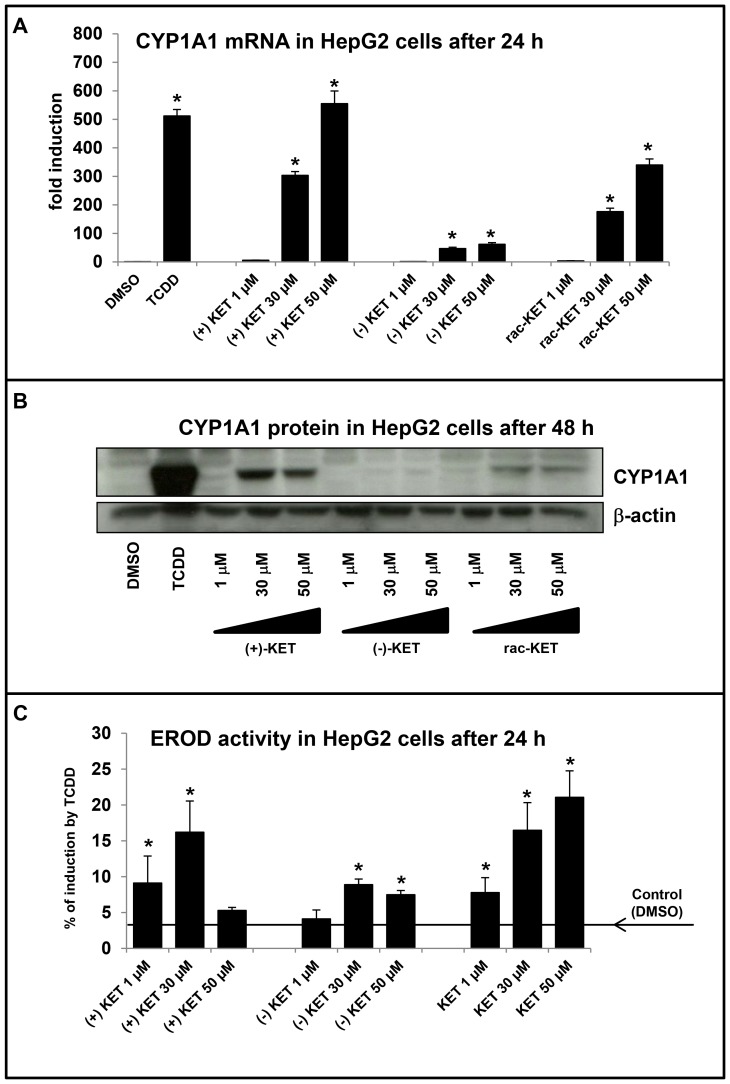
Effect of ketoconazole enantiomers on CYP1A1 mRNA, protein and EROD activity in human cancer cell line HepG2. HepG2 cells were seeded in 6-well plates and stabilized for 16 h. All experiments were performed in three independent cell passages. Cells were incubated for 24 h (mRNA and EROD analysis) or 48 h (protein analysis) with (+)-KET, (−)-KET and commercial rac-KET at concentrations 1 µM, 30 µM and 50 µM. **Panel A and Panel B:** Representative RT-PCR analysis of CYP1A1 mRNA and western blot of CYP1A1 protein are showed (data from passage #2). RT-PCR data are the mean ± SD from triplicate measurements and are expressed as fold induction over vehicle-treated cells. The data were normalized to GAPDH mRNA levels. **Panel C:** CYP1A1 activity (7-ethoxyresorufin-O-deethylase; EROD) was measured by spectrofluorometry with 530 nm excitation and 590 nm emission filters. Treatments were performed in triplicates. Average EROD data from three independent passages are showed. Data are expressed as the percentage of TCDD-induced activity. An asterisk (*) indicates that the value is significantly different from the activity of TCDD.

In next series of experiments, we tested the ability of KET enantiomers to induce the expression of prototypical AhR-responsive gene - CYP1A1. Human hepatoma HepG2 cells were treated with TCDD (5 nM), vehicle (DMSO; 0.1% V/V), rac-KET, (+)-KET and (−)-KET at concentrations 1 µM, 30 µM and 50 µM for 24 h (mRNA expression, EROD activity) and 48 h (protein expression). Dioxin, a model activator of AhR and inducer of CYP1A1 induced CYP1A1 mRNA approximately 500-fold as compared to vehicle-treated cells. All forms of KET, i.e. commercial racemic rac-KET, (+)-KET and (−)-KET displayed a concentration-dependent increase of CYP1A1 mRNA level. In line with the data from the AhR gene reporter assays, the strongest induction of CYP1A1 mRNA was achieved by (+)-KET at all concentrations tested (induction by 50 µM was comparable with 5 nM TCDD), while (−)-KET displayed approx. 10% of induction by (+)-KET (Figure 2A). Commercial rac-KET showed the combined effect of (+)-KET and (−)-KET enantiomers (Figure 2A). Consistently with mRNA results, (−)-KET caused a very moderate induction of CYP1A1 protein, while (+)-KET strongly induced CYP1A1 protein with the maximum induction at concentration of 30 µM (Figure 2B). Commercial rac-KET caused higher induction of CYP1A1 protein than (−)-KET but significantly weaker in comparison with (+)-KET (Figure 2B). We also tested a capability of (+)-KET, (−)-KET and rac-KET to induce catalytic activity of CYP1A1 in HepG2 cells (EROD assay). Cells were treated for 24 h with tested compounds at concentrations 1 µM, 30 µM and 50 µM, with 5 nM TCDD or vehicle (DMSO; 0.1% v/v). TCDD induced EROD activity with the average increase of 22–23-fold. Rac-KET increased the EROD activity in a concentration-dependent manner with the maximum at 50 µM. (+)-KET and (−)-KET reached the maximal EROD induction at concentration 30 µM and the increase was 15% and 8% of TCDD potency, respectively (Figure 2C). Since EROD activity measured in cell culture comprise both the effect on catalytic activity and induction/repression of CYP1A1 gene, the data obtained must be interpreted with prudence. Collectively, the effects of KET on CYP1A1 mRNA and protein expression in HepG2 cells were enantiospecific.

### Effect of ketoconazole enantiomers on CYP1A1/2 mRNA, protein and EROD activity in primary human hepatocytes

Since KET induced CYP1A1 mRNA and protein in human hepatoma cells HepG2, we examined a capability of KET to induce CYP1A1 and CYP1A2 mRNA and protein in primary human hepatocytes, a more physiological and metabolically competent cell model. Human hepatocytes were treated for 24 h or 48 h with TCDD (5 nM), vehicle (DMSO; 0.1% V/V), rac-KET, (+)-KET or (−)-KET at concentrations 1 µM, 30 µM and 50 µM.

**Figure 3:**
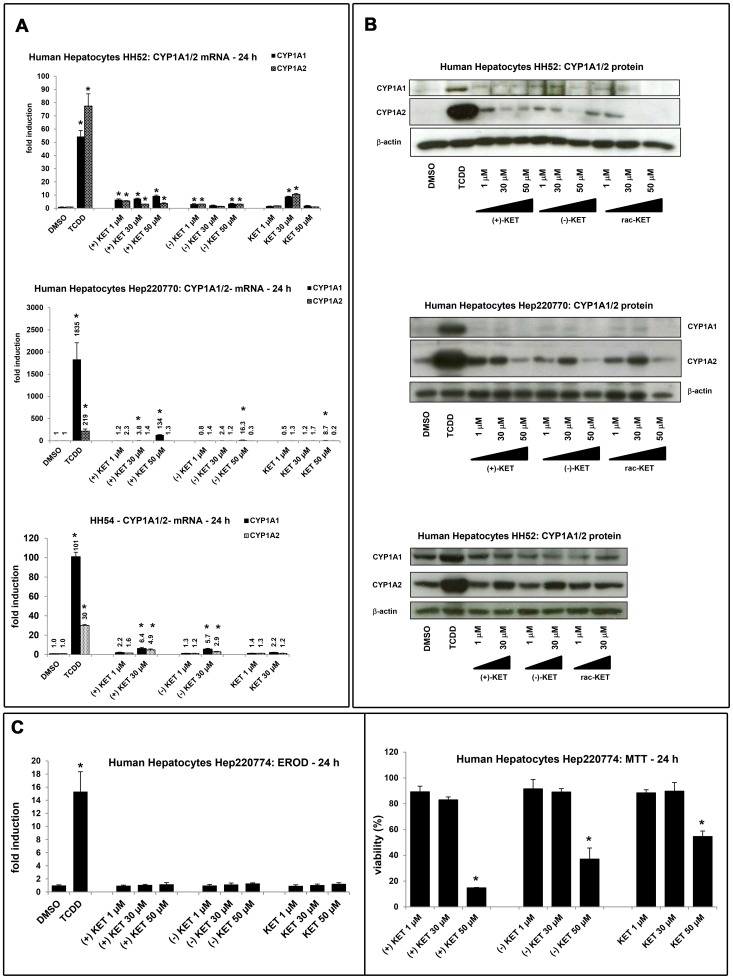
Effect of ketoconazole enantiomers on CYP1A mRNA, protein and EROD activity in primary human hepatocytes. Panel A: RT-PCR analyses CYP1A1 and CYP1A2 mRNA: Human hepatocytes were incubated for 24 h with (+)-KET, (−)-KET and commercial rac-KET at concentrations 1 µM, 30 µM and 50 µM. Results from three different cultures (HH52, HH54, Hep220770) are shown. Data are the mean ± SD from triplicate measurements and are expressed as fold induction over vehicle-treated cells. Data were normalized to GAPDH mRNA levels. **Panel B:** CYP1A1 and CYP1A2 protein analyses: Human hepatocytes were incubated for 48 h with (+)-KET, (−)-KET and commercial rac-KET at concentrations 1 µM, 30 µM and 50 µM. Western blots from three different cultures (HH52, HH54, Hep220770) are shown. **Panel C:** EROD and cytotoxicity: Human hepatocytes (culture Hep220774) were treated with (+)-KET, (−)-KET and commercial rac-KET at concentrations 1 µM, 30 µM and 50 µM. ***Upper bar graph:*** An activity of 7-ethoxyresorufin-*O*-deethylase (EROD) was measured by fluorescent spectrophotometry with 530 nm excitation and 590 nm emission filters. Treatments were performed in triplicates. The data are expressed as fold induction over the value from control cells. ***Lower bar graph:*** A conventional MTT test was performed and absorbance was measured at 540 nm. Treatments were performed in triplicates. The data are expressed as percentage of viability of control cells.

Induction of CYP1A1/CYP1A2 mRNAs by 5 nM TCDD in three different human hepatocytes cultures was 53-fold/79-fold (for HH52), 1835-fold/219-fold (for Hep220770) and 101-fold/30-fold (for HH54). Induction profiles by KET enantiomers varied between individual human hepatocytes cultures. In culture Hep220770, CYP1A1 mRNA was strongly induced by (+)-KET (134-fold; 50 µM), and weakly by (−)-KET (16-fold; 50 µM) and rac-KET (9-fold; 50 µM), while there was no induction of CYP1A2 mRNA by any form of KET. In culture HH54, both CYP1A1 and CYP1A2 mRNAs were induced by (+)-KET and (−)-KET, but not by rac-KET. The effects of KET enantiomers were similar (3-fold to 6-fold), and the inductions of CYP1A1 mRNA and CYP1A2 mRNA were comparable. In culture HH52, the effects of (+)-KET on CYP1A1 mRNA were a bit stronger as compared to (−)-KET or rac-KET. The induction of CYP1A2 mRNA by all forms of KET was equipotent (Figure 3A). Overall, while all forms of KET induced CYP1A genes in primary human hepatocytes, there were no significant enantiospecific effects, and the induction profiles varied between human hepatocytes cultures.

We found very faint or no induction of CYP1A1 protein after the treatment with any form of KET, while TCDD caused drastic increase of CYP1A1 protein in all three primary human hepatocytes cultures (HH52, HH54, Hep220770) (Figure 3B). On the other hand, dose-dependent induction of CYP1A2 protein was observed for all KET forms (Figure 3B): (i) at 1 µM concentration, the magnitude of induction was highest for (+)-KET as compared to (−)-KET and rac-KET; (ii) at 30 µM concentration, the induction by all KET forms was equipotent; (iii) at 50 µM concentration, the expression of CYP1A2 protein dropped, probably due to the cytotoxic effects of KET in human hepatocytes (*vide infra*).

Next, we tested capability of ketoconazole to induce catalytic activity of CYP1A1/1A2 enzymes and cytotoxicity in primary human hepatocytes. The cells were treated for 24 h with (+)-KET, (−)-KET and commercial rac-KET (1 µM, 30 µM, 50 µM), and with TCDD (5 nM) and vehicle (DMSO; 0.1% V/V). TCDD caused the induction of EROD activity approximately 15-fold while no significant induction of EROD activity was observed for any tested form of KET (Figure 3C). In the cytotoxicity assay, we found all forms of KET highly toxic against human hepatocytes at the concentration 50 µM. (+)-KET, (−)- KET and rac-KET decreased viability down to 15%, 37% and 46% of control value, respectively (Figure 3C).

Collectively, CYP1A1 and CYP1A2 genes were induced by KET in human hepatocytes. The induction profiles displayed inter-individual variability, and inconsistency between mRNA and protein expression was observed.

### AhR transformation and DRE binding by ketoconazole enantiomers

**Figure 4:**
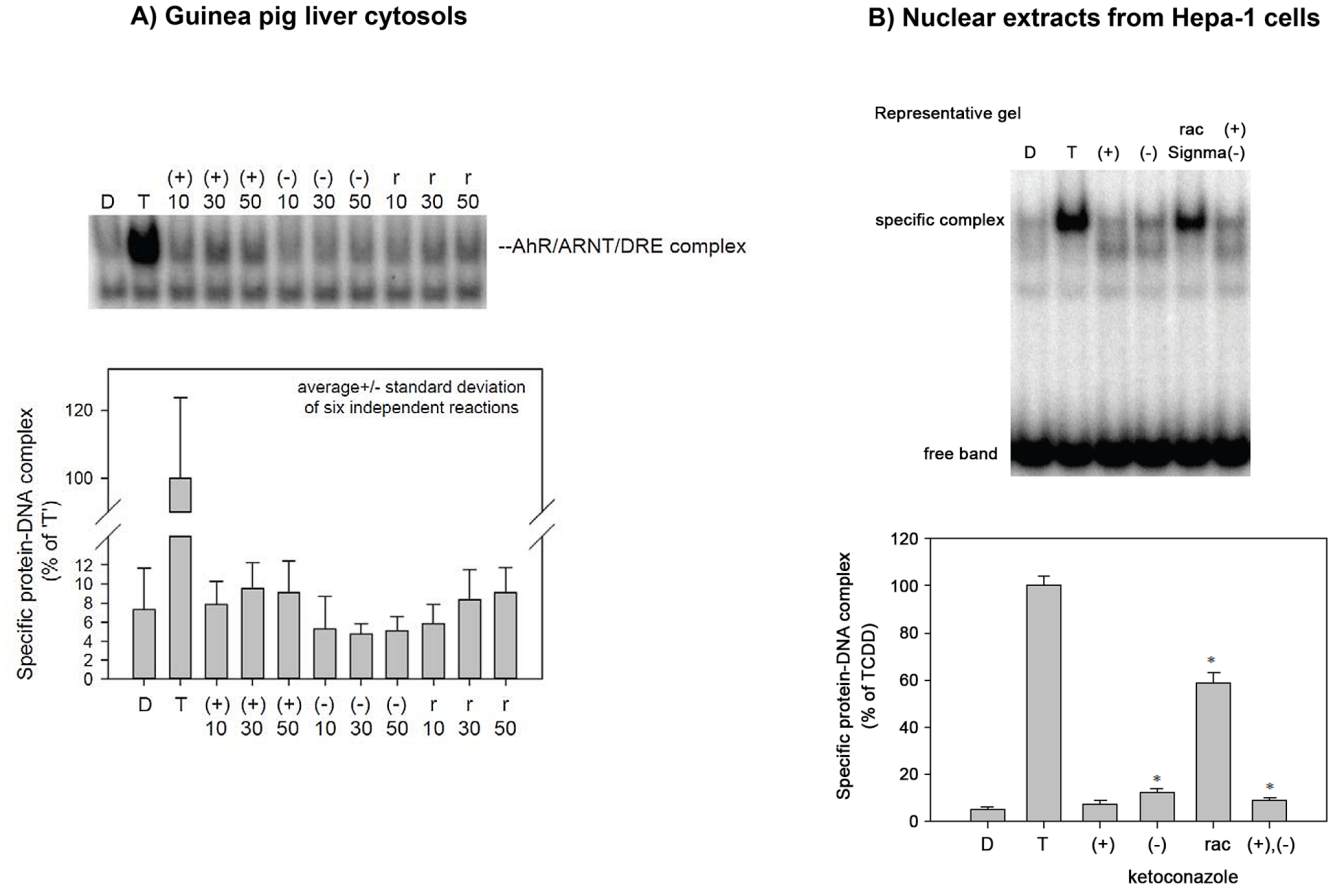
AhR transformation and DRE binding by ketoconazole enantiomers. Panel A: Electromobility shift assay EMSA with guinea pig hepatic cytosolic extract. Guinea pig cytosolic extract diluted to 8/mL protein in HEDG was incubated in the presence of 2% v/v DMSO (lane 1), 20 nM TCDD (lane 2), 50 µM (+)-KET (line 3), 50 µM (−)- KET (line 4), 50 µM rac-KET purchased from Sigma (line 5) for 1.5 h at room temperature and DNA binding was analyzed by GRA as described (Soshilov & Denison, 2014). A representative gel is shown. The bottom panel: Quantitation of the experiment showed in panel (A). Values represent the means ± SD of three independent experiments. **Panel B:** **EMSA with nuclear extracts.** Mouse Hepa-1c1c7 cells were treated for 2 h with DMSO (line 1), 10 nM TCDD (line 2), 50 µM (+)-KET (line 3), 50 µM (−)- KET (line 4), 50 µM rac-KET purchased from Sigma (line 5) and an equimolar mix of (+) and (−) enantiomers at final 50 µM total (line 6). The nuclear extracts were prepared and analyzed for DNA binding as described (Soshilov & Denison, 2014). A representative gel is shown. The bottom panel: Quantitation of the experiment shown in part (B). Values represent the means ± SD of three independent experiments.

We carried out the electrophoretic mobility shift assay (EMSA) to reveal whether effects of KET on AhR-CYP1A pathway involve transformation of AhR to DNA binding form, and whether these effects are enantiospecific. For this purpose, EMSA was performed in two experimental systems: (i) Hepatic guinea pig cytosol was treated for 2 hours with DMSO, 20 nM TCDD, (+)-KET, (−)-KET and commercial rac-KET (from Sigma) at 10 µM, 30 µM and 50 µM. All forms of KET transformed AhR to DNA binding form, but the activation was very weak and not statistically above background. There was a slightly stronger effect of (+)-KET and rac-KET as compared to (−)-KET, but the difference was not significant (Figure 4A). (ii) Since we obtained only weak signals in guinea pig liver cytosols, we also analyzed nuclear extracts from mouse hepatoma Hepa-1c1c7 cells treated for 2 hours with DMSO, TCDD (10 nM), (+)-KET (50 µM), (−)-KET (50 µM), commercial rac-KET (Sigma; 50 µM) or an equimolar mix of (+)/(−) enantiomers (50 µM total). Similarly as in guinea pig liver cytosol, (+)-KET and (−)-KET only weakly transformed AhR to DNA binding form. The effects of (−)-KET were slightly stronger as compared to (+)-KET. Surprisingly, commercial racemic KET strongly induced formation of AhR-ARNT-DRE complex (approx. 60% of TCDD effects), while racemic KET obtained by mixing pure (+)-KET and pure (−)-KET in ratio 1∶1, displayed the effects much weaker and comparable with individual enantiomers (Figure 4B). These results suggest the presence of impurities in commercial KET that can activate the mouse AhR more strongly than KET-enantiomers [20]. In addition, we observed formation of the second band with lower molecular weight than AhR-ARNT-DRE, by purified KET enantiomers but not by TCDD and commercial KET. Overall, the capability of ketoconazole enantiomers to induce a formation of AhR-ARNT-DRE complex does not seem to be enantiospecific, and the effects are much weaker than expected with regard to the magnitude of CYP1A1 induction and AhR activation in HepG2 cells.

### Ligand binding assay

**Figure 5:**
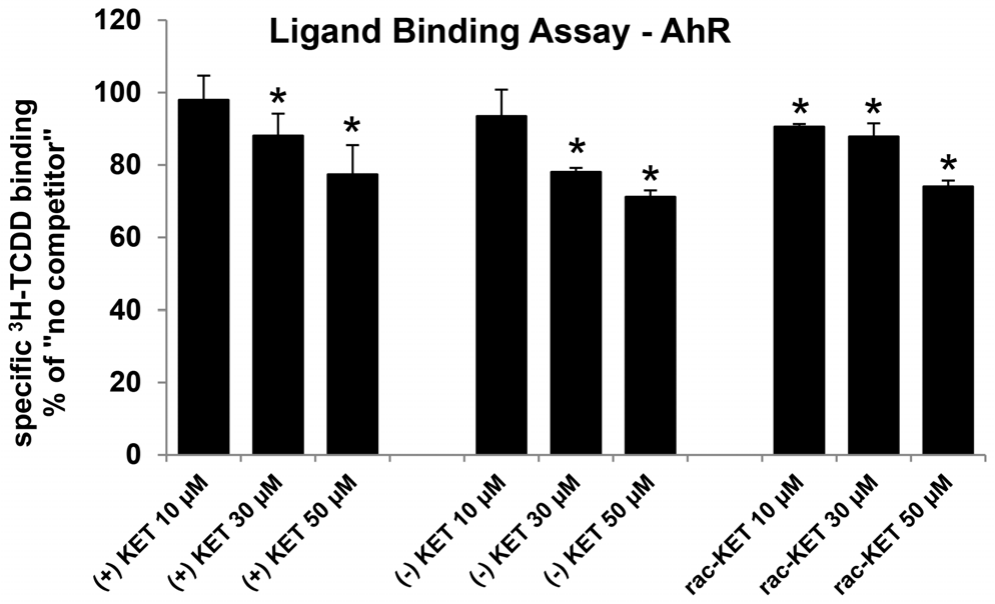
Ligand binding assay. Guinea pig hepatic cytosol was incubated with (+)-KET, (−)-KET and rac-KET (10 µM, 30 µM and 50 µM) or 200 nM TCDF for 1 h at room temperature in the presence of 2 nM [^3^H]-TCDD. Ligand binding to the cytosolic proteins was determined by the hydroxyapatite binding protocol and scintillation counting. Specific binding was determined as a difference between total and non-specific (TCDF) reactions. The values are presented as mean ± SD of three independent reactions. An asterisk (*) indicates that the value is significantly different from the ‘no competitor’ reaction at p<0.05 as determined by the Student's t-test. The results are representative of two independent experiments.

Since the activation of AhR may occur by ligand-dependent or ligand-independent mechanisms, we tested whether the effects of ketoconazole on AhR–CYP1A1 signaling pathway involve binding to the AhR. We performed AhR ligand binding assay using guinea pig hepatic cytosol. All tested forms of KET competitively, dose-dependently inhibited [^3^H]-TCDD binding to the AhR when present in the binding incubation at 30 µM and 50 µM (Figure 5). The differences between the effects of (+)-KET and (−)-KET enantiomers were not significant. Overall, both KET *cis*-enantiomers are weak AhR ligands their effects on AhR-CYP1A1 signaling pathway occur *via* ligand-dependent mechanism. The binding of KET to AhR was not enantiospecific.

### Effects of ketoconazole enantiomers on transcriptional activity of glucocorticoid receptor

**Figure 6:**
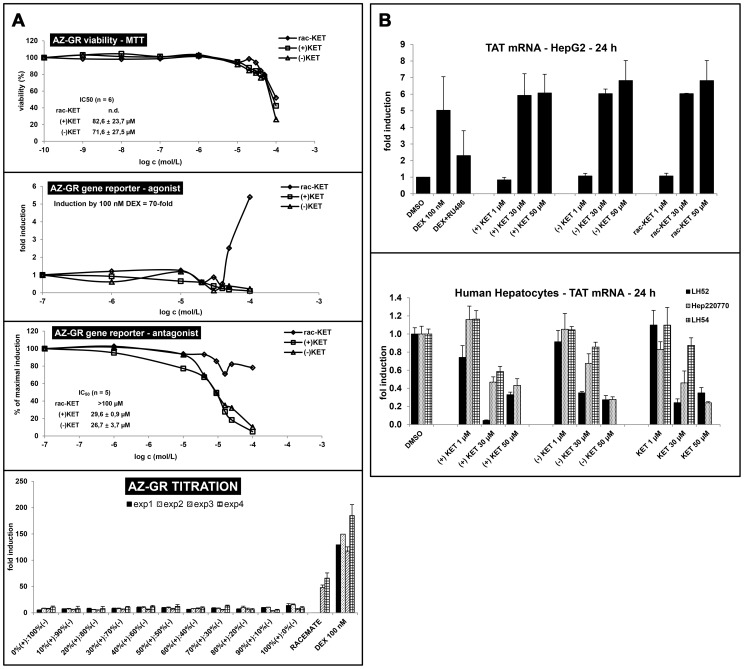
Effects of ketoconazole enantiomers on transcriptional activity of glucocorticoid receptor. Panel A: AZ-GR cells were seeded in 96-well plates, stabilized for 16 h and then incubated for 24 h with (+)-KET, (−)-KET, commercial rac-KET, DEX (100 nM) and vehicle (DMSO, 0.1% v/v). Treatments were performed in triplicates. The data are the mean from experiments performed in four different passages of cells. ***Upper plot:*** Cytotoxicity assay. Data are expressed as a percentage of viability of control cells; ***Middle plots:*** Representative gene reporter assays in AZ-GR cells – agonist/antagonist mode. Data are expressed as a fold induction of luciferase activity over control cells (*agonist mode*) or as a percentage of maximal induction attained by DEX (*antagonist mode*). ***Lower bar graph:*** AZ-GR cells were treated for 24 h with combination with (+)-KET and (−)-KET in ratios ranging from 0% (+): 100% (−) to 100% (+): 0% (−) at final concentration 50 µM, in the presence of DEX (100 nM). Data from four independent passages of cells are showed. Data are expressed as a percentage of maximal induction attained by DEX. **Panel B:** ***Upper bar graph:*** HepG2 cells were seeded in 6-well plates and stabilized for 16 h. Cells were incubated for 24 h with DEX (100 nM), DEX+RU486, (+)-KET, (−)-KET and commercial rac-KET at concentrations 1 µM, 30 µM and 50 µM. Experiments were performed with three independent cell passages. Representative RT-PCR analysis of TAT mRNA is showed. ***Lower bar graph:*** Human hepatocytes were incubated for 24 h with (+)-KET, (−)-KET and commercial rac-KET at concentrations 1 µM, 30 µM and 50 µM. RT-PCR analyses of tyrosine aminotransferase TAT mRNA from three different cultures (Hep220770, LH52, LH54) are showed. RT-PCR data are the means ± SD from triplicate measurements and are expressed as fold induction over vehicle-treated cells. The data were normalized per GAPDH mRNA levels.

There is multiple evidence for the role of glucocorticoid receptor (GR) in regulation of AhR activity [21]. Therefore, we tested whether the effects of KET on AhR involve GR. For this purpose, we incubated gene reporter cell line AZ-GR for 24 h (+)-KET, (−)-KET and rac-KET [18]. All forms of KET displayed cytotoxic effect in AZ-GR cell line without significant enantiospecificity, as assessed by MTT test. The values of IC_50_ were 82.6±23.7 µM for (+)-KET and 71.6±27.5 µM for (−)-KET. The IC_50_ value for rac-KET was higher than 100 µM (Figure 6A). Gene reporter assays were performed in *agonist* and *antagonist mode* (similarly as described in section 3.1.). In *agonist mode*, cells were incubated with increasing concentrations of (+)-KET, (−)-KET and commercial rac-KET and with model GR agonist (DEX; 100 nM). An induction of GR-dependent luciferase activity by 100 nM DEX in four independent passages of AZ-GR cells varied from 45-fold to 85-fold (average induction 70-fold), as compared to vehicle-treated cells. *Cis*-enatiomers (+)-KET and (−)-KET did not induce luciferase activity in AZ-GR cells up to maximal applied concentration, i.e. 100 µM. Interestingly, approximately 5-fold increase of luciferase activity was observed for commercial rac-KET at concentration 100 µM (Figure 6A). In *antagonist* mode, (+)-KET and (−)-KET showed strong antagonist effect on GR transcription activity, and there was no significant difference between the effects of individual enantiomers. The values of IC_50_ were 29.6±0.9 µM and 26.7±3.7 µM for (+)-KET and (−)-KET, respectively. We observed only weak inhibition of DEX-induced luciferase activity by commercial rac-KET (Figure 6A). To further elucidate the discrepancy between the effects of pure enantiomers as compared to commercial racemic KET, we performed *titration experiment* in antagonist mode to examine the effect of (+)- and (−)-KET on GR transcription activity. The AZ-GR cells were treated for 24 h with DEX (100 nM) in combination with mixtures of pure (+)-KET and pure (−)-KET, in ratios from 0% (+): 100% (−) to 100% (+): 0% (−), at final concentration 50 µM. Antagonist effect by mixture of 50% (+)-KET: 50% (−)-KET was much stronger as compared to commercial rac-KET (Figure 6A). The plausible explanation could be the influence of impurities contained in commercial rac-KET, similarly as observed in EMSA experiments.

We also analyzed the expression of tyrosine aminotransferase (TAT), the GR-target gene, in cell line HepG2 and in primary human hepatocytes. In HepG2 cells, we found strong, concentration-dependent induction of TAT mRNA by (+)-KET, (−)-KET and rac-KET. The magnitude of induction by 30 µM and 50 µM KET was comparable with effects of DEX, and the effects of KET were not enantiospecific (Figure 6B). Primary human hepatocytes are routinely cultured in the presence of DEX, therefore, the TAT gene is induced under these conditions. All forms of KET strongly, and dose-dependently down-regulated TAT mRNA in three different primary hepatocytes cultures Hep220770, HH52 and HH54. Again, the effects of KET were not enantiospecific. Overall, KET displayed strong, but inconsistent and enantio non-specific agonist and/or antagonist effects on GR in various *in vitro* cell systems (Figure 6A, Figure 6B).

### Antifungal activity of ketoconazole enantiomers

Ketoconazole is the antimycotic agent, clinically used as a racemic mixture of *cis*-enantiomers. Therefore, we tested antifungal activity of separate enantiomers and commercial racemate in *Candida spp*. strains: *C. albicans, C. krusei, C. tropicalis and C. parapsilosis*. Antifungal activity of (+)-KET, (−)-KET and commercial rac-KET against clinically important fungi is summarized in Table 1 and the data for currently used antifungals (voriconazole, posaconazole, fluconazole) are showed in Table 2. (+)-KET was two times more potent than (−)-KET for strains *C. albicans* and *C. tropicalis*, while (−)-KET was seven times more potent than (+)-KET for other tested microorganisms. Hence, we demonstrate enantiospecific antifungal activity of ketoconazole, which is in line with observations of other authors [7].

**Table 1:** Activity of ketoconazole enantiomers (in µM).

Candida spp.	(+) KETOCONAZOLE	(−) KETOCONAZOLE	(rac) KETOCONAZOLE
*C. albicans* 1	25	50	50
*C. krusei* 3	1.56	0.20	1.56
*C. tropicalis* 5	3.13	6.25	6.25
*C. parapsilosis* 6	1.56	1.56	1.56
*C. albicans 978*	25	50	50
*C. krusei 094*	1.56	0.20	1.56

MIC/MFC - minimum fungicidal concentration was the same as minimum inhibitory concentration in all cases.

**Table 2:** MIC values for fluconazole, voriconazole and posaconazole against the tested *Candida* spp. strains (mg/L).

Candida spp.	Posaconazole	Voriconazole	Fluconazole
*C. albicans* 1	0.094	0.016	0.38
*C. krusei* 3	0.190	0.190	48
*C. tropicalis* 5	0.190	0.190	6
*C. parapsilosis* 6	0.032	0.023	2
*C. albicans 978*	0.064	0.016	1.5
*C. krusei 094*	0.250	0.190	32

## Discussion

The effects of KET on drug-metabolizing pathways are very complex, and involve inhibition of cytochrome P450 catalytic activities (e.g. CYP3A4, CYP3A5, CYP2C9), agonism and/or antagonism of several receptors, including glucocorticoid receptor (GR) [12], aryl hydrocarbon receptor (AhR) and pregnane X receptor (PXR) [22], or interactions with drug transporters [13]. With regard to AhR, azole antifungal drugs were shown to influence AhR-dependent genes in aquatic species [23], [24], rodents [25] and in murine and human cancer cell lines [14]. Clinically used KET is a mixture of two *cis*-enatiomers (2R,4S)-(+)-KET and (2S,4R)-(−)-KET. Enantiospecific effects of KET on catalytic activities of CYP3A4/5 [8] and progesterone 17α, 20-lyase were reported [7]. A phase II clinical study was conducted with compound DIO-902 (which is ketoconazole enantiomer (−)-KET), as a candidate drug for the treatment of Diabetes mellitus Type II [26]. The mechanism of DIO-902 action was enantiospecific inhibition of cortisol synthesis. However, due to the side effects, a study was interrupted and DIO-902 was suspended [27]. In the current paper, we investigated enantiospecific effects of KET on AhR-CYP1A signaling pathway in cancer cell lines and in human hepatocytes, and we provide the first evidence of enantiospecific interactions of KET with AhR-signaling pathway *in vitro*. In particular, we demonstrate that:

- KET activates AhR in gene reporter cell line and dose-dependently induces CYP1A1 mRNA and CYP1A1 protein in HepG2 cells, with enantiospecific pattern, i.e. (+)-KET was much more active as compared to (−)-KET.
- KET enantiospecifically induces CYP1A1 and CYP1A2 mRNA and CYP1A2 protein in primary cultures of human hepatocytes. The effects of enantiomer (+)-KET were stronger than those of (−)-KET enantiomer.
- Both KET enantiomers are weak ligands for AhR, as revealed by ligand binding assay performed in guinea pig liver cytosols. In addition, both enantiomers of KET transformed AhR to DNA-binding form, as revealed by EMSA assays in guinea pig liver cytosols and Hepa-1c1c7 murine cells. Neither binding to AhR nor transformation of AhR to DNA-binding form was enantiospecific. Interestingly, we observed that commercial racemic KET strongly induced formation of AhR-ARNT-DRE complex (approx. 60% of TCDD effects), while in mouse but not guinea pig the racemic KET obtained by mixing pure (+)-KET and pure (−)-KET in ratio 1∶1, displayed the effects much weaker and comparable with individual enantiomers. Strong effects of commercial racemic KET are probably caused by the impurities present in commercial product [20]. This implies that many *in vitro* effects of KET described in scientific literature may be due to the presence of impurities, and these data should be interpreted with prudence.
- KET is strong antagonist of human glucocorticoid receptor GR, as revealed by gene reporter assays in transgenic AZ-GR cells and by down-regulation of tyrosine aminotransferase TAT in primary human hepatocytes cultured in dexamethasone-containing medium. We also observed induction of TAT in HepG2 cells by KET, suggesting partial agonist and antagonist effects of KET. The effects of KET on GR were not enantiospecific, implying no role for GR in enantiospecific induction of CYP1A1 and activation of AhR. This is an important finding, taking in account a pivotal role of GR in regulation of AhR transcriptional activity [21]. Racemic commercial KET showed irregular behavior, suggesting the influence of impurities, similarly as in EMSA experiment.
- Enantiospecific antifungal activity of KET was observed in several *Candida spp*. strains including *C. albicans 1*, *C. albicans 978*, *C. krusei 3*, *C. krusei 094* and *C. tropicalis 5*, which is also in line with observations of other authors [7].

In conclusion, current study provides the first evidence of enantiospecific effects of ketoconazole on AhR signaling pathway. The results might have clinical significance since (+)-KET activates and induces CYP1A1, but (−)-KET has higher antifungal activity in some Cancida spp. strains.
